# Traumatic Vertebral Artery Dissection Without Cervical Spine Injury Treated by Mechanical Thrombectomy

**DOI:** 10.7759/cureus.60287

**Published:** 2024-05-14

**Authors:** Hiroki Namikawa, Atsushi Ogata, Takashi Furukawa, Jun Masuoka, Tatsuya Abe

**Affiliations:** 1 Department of Neurosurgery, Saga University, Saga, JPN

**Keywords:** cerebral infarction, traffic trauma, basilar artery occlusion, mechanical thrombectomy, traumatic vertebral artery dissection

## Abstract

A 59-year-old female injured in a motor vehicle accident presented with progressively impaired consciousness, and emergent magnetic resonance imaging (MRI) revealed basilar artery occlusion (BAO). Mechanical thrombectomy (MT) was performed immediately and achieved complete recanalization. Contrast-enhanced MRI also indicated right vertebral artery dissection (VAD), and the patient was subsequently diagnosed with artery-to-artery embolism caused by traumatic VAD. Anticoagulation therapy was initiated postoperatively, and there was no VAD or BAO recurrence during the three-month follow-up. This is the first reported case of BAO caused by traumatic VAD in an adult without accompanying cervical vertebral fracture treated using MT.

## Introduction

Traumatic vertebral artery dissection (VAD) is a rare complication of blunt head and neck trauma but is associated with a high rate of cerebral infarction [1]. While mechanical thrombectomy (MT) is widely performed for large vessel occlusion (LVO), there are very few reports of MT use for basilar artery occlusion (BAO) secondary to traumatic VAD [2-7]. Further, all previous adult cases of VAD treated using MT were complicated with cervical spine fracture [2,3,5,6], and there are only two published pediatric cases without cervical spine fracture [4,7]. Thus, this is the first report of MT use for BAO due to traumatic VAD in an adult without cervical spine fracture.

## Case presentation

**Figure 1:**
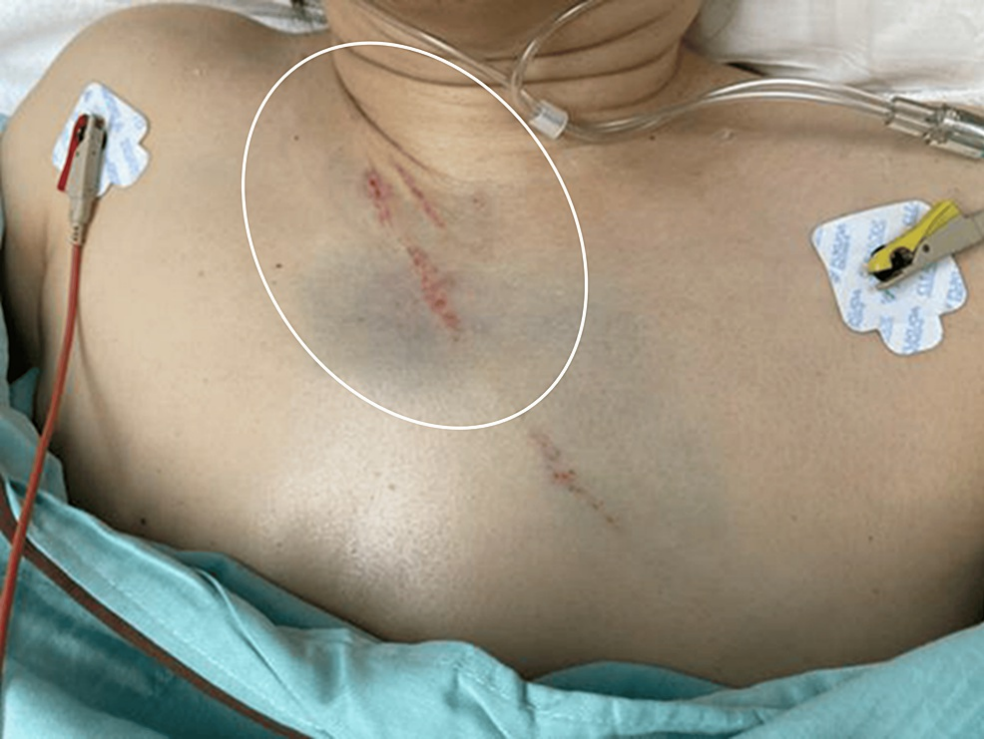
Photograph of the anterior chest Seat belt indentation on the anterior chest of the patient (white circle) following an automobile accident.

**Figure 2:**
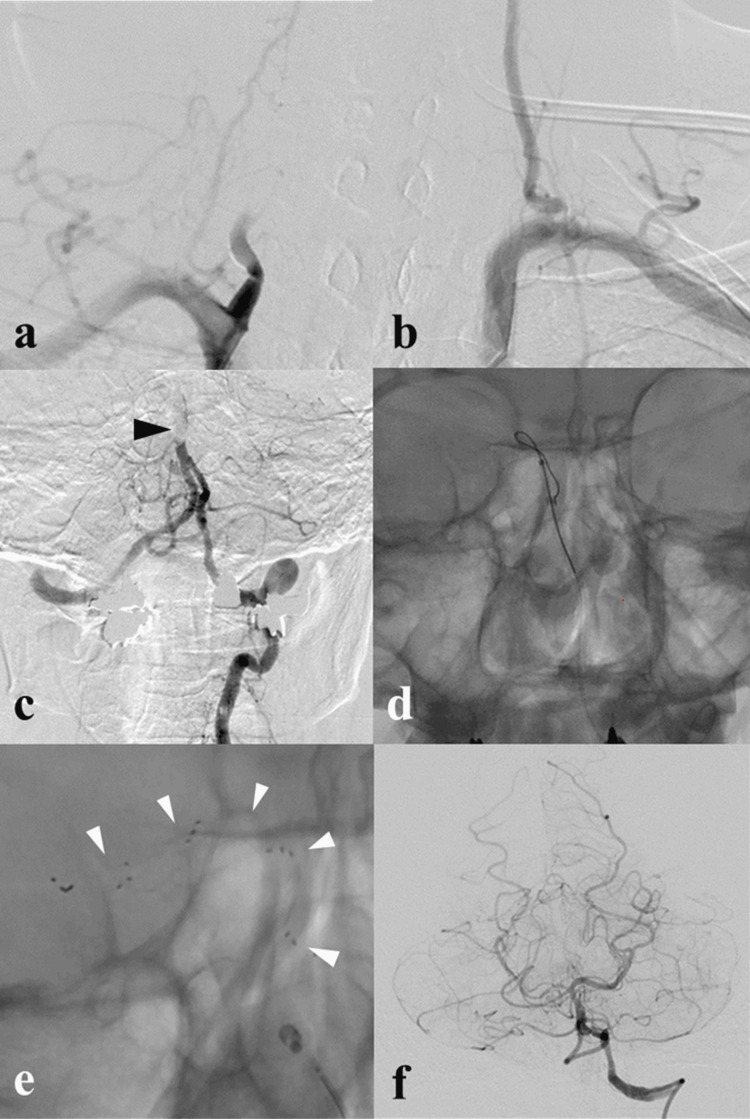
Imaging findings in the mechanical thrombectomy (a) Right brachiocephalic angiogram showing occlusion of the right vertebral artery (VA). (b) Left subclavian angiogram showing an intact left VA. (c) Left vertebral angiogram showing occlusion of the basilar artery (BA, black arrowhead). (d) X-ray image showing a micro guidewire passed through the occlusion. (e) X-ray image showing the stent retriever deployed from the posterior cerebral artery (PCA) to BA (white arrowhead). (f) Left vertebral angiogram showing complete BA recanalization after mechanical thrombectomy (MT).

**Figure 3:**
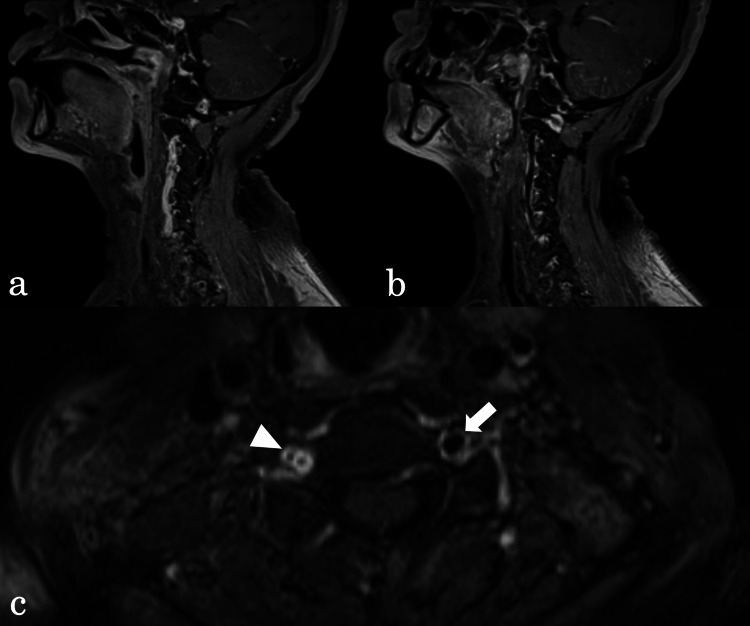
Contrast-enhanced T1-weighted MRI (a) The right vertebral artery (VA) showing dissection. (b) The left VA showing normal findings. (c) In contrast to the normal left VA (white arrow), the right VA (white arrowhead) showed double-barrel findings due to dissection.

**Figure 4:**
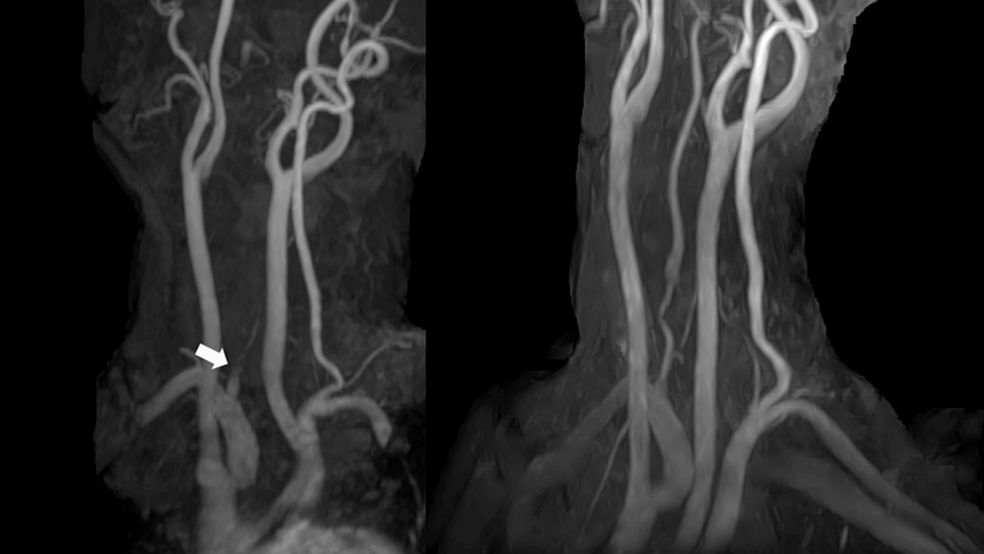
Vascular imaging findings following MT (a) Neck magnetic resonance angiography (MRA) on the day after MT showing the occluded right VA (white arrow). (b) Neck MRA three months after onset showing recanalization of the right VA.

A 59-year-old female was transported to the emergency room with injuries sustained in an automobile accident. She had the imprint of a seatbelt on her anterior chest and neck pain (Figure 1). There was no impairment of consciousness or neurologic deficit. Whole-body non-contrasted computed tomography (CT) revealed no obvious traumatic injuries, including no cervical spine injuries. After admission, however, dizziness and nausea appeared, followed by restlessness and disorientation. Approximately nine hours after injury, her consciousness level had decreased to Glasgow Coma Scale (GCS) grade E2V4M5, and left hemispatial neglect appeared, but there was no apparent motor paralysis. At that time, the National Institute of Health Stroke Scale (NIHSS) score was 12. Initially, the symptoms were thought to be due to delirium, but the severe impairment of consciousness and focal neurologic signs suggested the possibility of cerebrovascular disease. A non-contrast head CT scan showed a high-density area in the right vertebral artery (VA). Magnetic resonance angiography (MRA) revealed basilar artery occlusion (BAO), and diffusion-weighted magnetic resonance imaging (DWI) revealed acute infarcts in the right cerebellum and right thalamus. Further, emergent cerebral digital subtraction angiography (DSA) showed that the right VA was occluded from its origin (Figure 2a), but the left VA was intact (Figure 2b). Therefore, we introduced a 6Fr FUBUKI Dilator Kit 80cm guide catheter (ASAHI INTECC, Nagoya, Japan) into the left (contralateral) VA and attempted to perform a thrombectomy to address the BAO (Figure 2c). In the first pass, we guided a Catalyst6 aspiration catheter (Stryker, Fremont, CA) to the occluded portion of the BA for attempted direct aspiration first-pass technique (ADAPT), but recanalization failed. In the second pass, we guided a Phenom27 microcatheter (Medtronic, Minneapolis, MN) into the right posterior cerebral artery (PCA) (Figure 2d), deployed a Solitaire X 6mm/40mm stent retriever (Medtronic, Minneapolis, MN) from the right PCA to BA (Figure 2e), and carefully retrieved the Solitaire X into the Catalyst6 by continuous aspiration. After retrieval, DSA confirmed complete recanalization (Figure 2f). A long and soft red thrombus was attached to Solitaire X. In total, 238 min elapsed from onset to puncture and 31 min from puncture to recanalization. Postoperatively, consciousness improved to GCS E4V4M6, and a non-contrast head CT scan showed no hemorrhage. On the day after MT, DWI showed no obvious enlargement of the infarction. Contrast-enhanced T1-weighted magnetic resonance imaging (MRI) revealed the false lumen of the right VA (Figure 3). From these findings, we concluded that the stroke was caused by artery-to-artery embolism associated with traumatic VAD. We administered unfractionated heparin to prevent a recurrence and later changed to oral warfarin. Although slight dysarthria remained, the patient was transferred for rehabilitation on postoperative day 16 with a modified Rankin Scale (mRS) score of 1. No episodes of cerebral infarction occurred during follow-up, and the right VA was recanalized three months after the accident, as revealed by MRA (Figure 4). At that point, there was no neurologic deficit except for complaints of mild tinnitus.

## Discussion

Traumatic VAD occurs in only about 0.5% of blunt head and neck trauma cases but is complicated by cerebral infarction in 20% of this clinical subgroup [1]. Early diagnosis and treatment are important, and the screening criteria for blunt cerebrovascular injury (BCVI) (Table 1), also known as the Denver Criteria, may be useful for diagnosis [8]. Treatment strategy and device selection vary from case to case, although very few cases have been treated with MT. There are reports of patients treated with recombinant tissue plasminogen activator (rt-PA) in combination with MT [2,3], but the present case is unique for several reasons. First, the catheter was inserted into the contralateral VA, while other surgeons chose the ipsilateral side due to concerns that catheter insertion into the contralateral VA would reduce BA blood flow [4,5]. In addition, the CT scan showed no cervical spine fracture or dislocation despite the case patient presenting with cervical pain. In contrast, cervical spine injury is frequent in traumatic VAD (as high as 71%) [1] and has been observed in all cases of traumatic VAD reported [2,5,6] with the exception of two pediatric cases [4,7]. To date, there have been no published reports of traumatic VAD without cervical spine fracture in adults treated using MT. Various treatments have been used for the prevention of cerebral infarction after traumatic VAD. Some reports have suggested prior embolization of dissected vessels as prophylaxis against embolization in cases requiring cervical fracture repair [9,10]. In the current case, the patient did not need cervical spine repair, so we decided not to perform coil embolization. It is not clear whether antiplatelet agents or anticoagulants are more effective in preventing recurrent stroke in vertebral artery dissection [11]. Considering the trauma, heparin, which can be antagonized in an emergency, was considered appropriate. We initiated anticoagulation therapy with unfractionated heparin after the MT and later switched to oral warfarin. The patient continued warfarin treatment during follow-up without experiencing any hemorrhagic complications or recurrent embolisms. Additional case reports and series are needed to define the best treatment options for traumatic VAD cases with distinct clinical features.

**Table 1:** The screening criteria for blunt cerebrovascular injury (BCVI) The symptoms of focal neurologic defects and seat belt abrasion and altered mental status in the present case were also met the screening criteria.

Signs/Symptoms
Arterial hemorrhage from the neck, nose, and/or mouth
Expanding cervical hematoma
Cervical bruit in patient <50 years old
Focal neurologic defect (including Transient ischemic attack (TIA), hemiparesis, vertebrobasilar symptoms, Horner syndrome, etc.)
Ischemic stroke findings at CT or MR imaging
Neurologic deficit, inconsistent with head CT findings
Risk factors
High-energy mechanism with displaced midface fracture (Le Fort Ⅱ or Ⅲ), basilar skull fracture with carotid canal involvement, or Diffuse axonal injury and GCS <6
Cervical spine fracture or subluxation, including vertebral body fracture, transverse foramen fracture, subluxation or ligamentous injury at any level, or any fracture at C1 through C3
Near-hanging with anoxia
Clothesline-type injury or seat belt abrasion with swelling, pain, and/or altered mental status
Mandible fractures
Complex skull fractures
Scalp degloving
Thoracic vascular injuries
Traumatic brain injury with thoracic injuries

## Conclusions

Traumatic VAD may occur even in minor traumatic injuries without cervical spine fracture. Even if there is no neurological deficit and the trauma appears to be minor, screening for BCVI should be referred, and appropriate imaging studies, such as contrast-enhanced CT, should be added. Early diagnosis is difficult if the patient is asymptomatic. Even when symptoms do appear, if they resemble delirium, as in this case, the diagnosis is even more difficult. If the patient presents with restlessness or disorientation shortly after trauma, it may be better to consider the possibility of this disease and perform a head MRI scan immediately. Based on case reports to date, MT is considered to be a safe and effective treatment for these cases and should be performed as soon as possible.
